# Reviews

**Published:** 1881-06

**Authors:** 


### Reviews.

---

**The Diseases of Children**; a Practical and Systematic work for Practitioners and Students. By WILLIAM HENRY DAY, M. D , Physician to the Samaritan Hospital for Women and Children. Second edition. Re-written and much enlarged. Philadelphia: Presley Blakiston, No. 1012 Walnut street 1881. Price, cloth, $5.00; sheep $6 00 Buffalo: Peter Paul & Bro.

Dr. Day will be at once recognized as the author of the valuable work, to which attention was directed in a previous number of this Journal, on headaches: their cause, nature and treatment. In the wider field here entered upon, the author demonstrates his familiarity with this department, and gives abundant evidence of a special aptitude for the investigation of the interesting class of diseases, observed in childhood. In this, he has had ample opportunities in the Samaritan Hospital, of which he is the attending physician, and he has improved them in a methodical and systematic manner, as is plainly evident in the work here offered to the profession. He emphasizes the position that the diseases of children should be considered separately and specially. We think every practitioner, whose attention has been much directed to the subject, will recall the inadequate instruction received before taking his degree in treating diseases peculiar to infancy and childhood. It is only by studying works of this character that defects of education can be remedied, and a familiarity and skill obtained in a department, that will constitute a large proportion of the work daily imposed upon him. Dr. Day here presents a valuable guide and a useful help in both study and practice. In many respects it is superior to West's or Smith's work. We commend it to the notice of the general practitioner, who is frequently called to combat the multifarious diseases peculiar to the early years of life.

---

**Lectures on Diseases of the Nervous System, especially in Children.** By S. WEIR MITCHELL, M. D.; with five plates. Philadelphia: Henry C. Lea's, Son & Co. 1881.

The present work of Dr. Mitchell is not unlike his previous contributions to the medical literature of the day, full of valuable information upon the subjects, of which it treats. He presents here thirteen lectures, and illustrates the subjects with clinical cases, gathered from his large experience. He treats such subjects as paralyses of hysteria, hysterical motor ataxia, mimicry of disease, unusual forms of spasmodic affections in women, tremor, chronic spasms, chorea of childhood, habit chorea, disorders of sleep in nervous or hysterical persons, etc., etc.

We have read the work with great interest and profit, and commend it to the profession as the most practical treatise of its kind.

---

**A Manual of the Practice of Medicine**; designed for the Use of Students and the General Practitioner. By HENRY C MOIR, M. D. New York: Steam-Press of the Industrial School H. O. A., 187 and 189 East Seventy-sixth street. 1881. Price, $2.50.

The author of this small compendium states that it comprises the substance of a course of lectures, given by a well-known instructor to his private pupils as a preparation for competitive examinations, and also embraces a careful résumé of several standard works. It presupposes some erudition upon the part of the reader, and is therefore adapted to the physician, who has but little leisure to examine exhaustive treatises. We have always questioned the utility of such works, believing that they tend to make superficial students, who use, in the hurry of the moment, the salient points of a case, and fail afterwards to examine deeply into its pathology.

---

**What Every Mother Ought to Know.** By EDWARD ELLIS, M. D. Philadelphia: Presley Blakiston, 1012 Walnut street. 1881. Price, 75 cents.

Dr. Ellis presents in this little book practical hints to young mothers for their guidance during pregnancy, and also for the management of the new-born. He emphasizes the principle that those who would be blessed with healthy children, must be healthy themselves. We think the work a very useful one.

---

**Constipation plainly treated and relieved without the Use of Drugs.** By JOSEPH F. EDWARDS, M. D. Philadelphia: Presley Blackiston, 1012 Walnut street. 1881. Price, 75 cents.

A small work, containing the author's opinions in regard to habitual constipation, and the measures necessary to overcome it. He submits it for "what it is worth," and inasmuch as the price is very low, it should evidently be worth the sum asked, if it is worth anything at all. The habit of constipation is a common one, especially among females. We hope the author has offered hints and advice to overcome it.

---

**Medical Diagnosis.** By J. M. DA COSTA, M. D. Philadelphia: J. B. Lippincott & Co. Fourth edition.

The former editions of this work are so well known that a review of its scope and object may well be considered as entirely unnecessary. Still the importance of the subject calls for a few remarks. It has often and truthfully been observed that the main difference in physicians, as regards rank and qualifications, is to be found in their varying skill in diagnosis. The disease, having been accurately diagnosed, it is a comparatively easy matter to determine the best known treatment. The work here noticed is therefore one whose study is of the first importance. That the author is equal to his task, no one can question, and when we add that the present edition appears to be in all respects fully up to date, we have clearly given all that is called for—an unreserved endorsement.

---

**Lectures upon Diseases of the Rectum and the Surgery of the Lower Bowel.** Delivered at the Bellevue Medical College, by W. H. VAN BUREN, M. D , Professor of the Principles and Practice of Surgery in the Bellevue Hospital Medical College, etc. New York: D. Appleton & Co , 1, 3 and 5 Bond street. 1881.

The lectures of Dr. Van Buren are well known and highly appreciated by the profession. This is, strictly speaking, a second edition of his book, but has been largely re-written and a great deal of new matter introduced, which makes it of still greater value. No troubles produce more misery and are oftener overlooked than the different diseases of the rectum, as for example, ulcers, fissures, piles, fistulas—and few can be more successfully relieved, if the physician is careful and painstaking in his examination.

We know of no book that covers the subject more completely than this.

---

**Medical Electricity; a Practical Treatise on the Applications of Electricity to Medicine and Surgery.** By ROBERT BARTHOLOW, M. D., Professor of Materia Medica and General Therapeutics in the Jefferson Medical College, etc. With ninety-six Illustrations. Philadelphia: Henry C. Lea's, Son & Co. 1881.

We have seen more voluminous works on medical electricity which very few physicians read and fewer understand, but we have not yet come across a book, that can compare with this in clearness and simplicity of statement. It is written for students and practitioners, and the author "has assumed an entire unacquaintance with the elements of the subject as the point of departure." We have, for a long time, needed a text-book on medical electricity, condensed and yet complete, and this want has been well supplied by the distinguished author. It is divided into six parts, electro-physics, electro-physiology, electro-diagnosis, electro-therapeutics, electricity in surgery and thermo-electricity, each of which does full justice to the subject. The illustrations are elegant, and the book as a whole is a valuable addition to the collection of any student or practitioner.

---

**Hernia, Strangulated and Reducible.** With Cure by Subcutaneous Injections, together with Suggested and Improved Methods for Kelotomy; also, an Appendix, giving a short Account of various New Surgical Instruments. By JOSEPH H. WARREN, M. D. With illustrations. Boston: Charles V. Thomas, 215 Tremont street. 1881.

The book with this voluminous title is an exposition of Dr. Heaton's method of treatment for the cure of hernia by injection, a method with which our readers are acquainted through Dr. Heath's paper in our April number. The author has improved the operation and perfected and invented new instruments, and he estimates his successful cures at 80—85 per cent. When properly performed, the operation is neither dangerous nor painful and deserves more attention than has heretofore been granted it.

---

**An Index of Comparative Therapeutics**; with Tables of Differential Diagnosis; a pronouncing list in the genitive case; a list of Medicines used in Homœopathic practice; Memoranda concerning Clinical Thermometry; Incompatibility of Medicines; Ethics; Obstetrics; Poisons; Anæsthetics; Fees; Asphyxia; Urinary Examinations; Homœopathic Pharmacology and Nomenclature, etc., etc. By SAMUEL O. L. POTTER, M. D., President, etc. Chicago: Duncan Brothers.

This book, as stated by the author, aims to present the therapeutics of the the two great medical schools in the manner best adapted to comparative study and ready reference. In parallel columns are placed the remedies recommended by the most eminent and liberal teachers in the regular and homœopathic branches of the profession. The work gives evidence of patient research, and of some learning on the part of its author, and we are, therefore, not surprised to read, that "Hahnemann advanced the most extreme and dogmatic tenets," and that "a few of his disciples followed his steps into the mystic realms of absurd speculation."

The book is well worth the price to both regular and homœopathic physicians.

---

**Practical Treatise on Diseases of the Skin..** By LOUIS A. DUHRING, M.D. Professor of Diseases of the Skin in the Hospital of the University of Pennsylvania, etc. Second edition; revised and enlarged. Philadelphia: J. B Lippincott & Co. 1881.

Since its appearance, five years ago, Duhring's Treatise on Diseases of the Skin has steadily grown in favor, and has gained an enviable reputation as a standard work on dermatology. The second edition has been carefully revised and enlarged, with reference to all the recent discoveries in this important branch of medical science. New chapters on certain obscure forms of skin-diseases, uridrosis, phosphorescent sweat, urticaria pigmentosa, etc., have been added. The nomenclature is the same as used by the standard authors, Hebra, Kapozi, Neuman, etc. No library is complete without this work, which, in connection with the author's Photographic Atlas of Skin Disease, will enable the student to follow the progress of dermatology up to the present day.

---

**The Metric System in Medicine;** containing an Account of the Metric System of weights; Measures Americanized and Simplified; a comprehensive Dose Table, etc., etc. By OSCAR OLDBERG, Phar. D, Medical Purveyor U. S. Marine Hospital Service, Prof. Materia Medica National College of Pharmacy, Washington, D. C., etc. Philadelphia: Presley & Blackiston. 1881. Price, cloth, $1.50.

This book is divided into three parts. Part I gives a very good description of the metric system, rules for conversion, tables of equivalents, and also a table of thermometric equivalents. Part II is entitled "Selected Prescriptions used in Hospital and out-patient practice," and contains 334 formulæ the quantities being expressed in metric terms, and the names of the drugs written with scientific defiance of classical rules. All this part of the book (77 pages) will be a grievance rather than a convenience to most readers. Part III is a pretty complete posological table, givingdoses in both apothecaries and metric terms.

---

**On the Antagonism between Medicines and between Remedies and Diseases;** being the Cartwright Lectures for the Year 1880. By ROBERT Bartholow, M. A., M. D., L. L. D., Professor of Matera Medica and General Therapeutics, in the Jefferson Medical College of Philadelphia, etc., etc. New York: D. Appleton & Co. 1881. Price, $1.25.

These lectures are the first fruits of the wise liberality of the late Mr. Cartwright, of Newark, N. J., whose bequest to the Alumni Association of the College of Physicians and Surgeons of New York, provides for an annual course of lectures and a prize essay. Such a gift, precious indeed on account of its rarity, encourages the growth of medical literature, stimulates the acquisition of new truths and offers to medical men an opportunity for honorable distinction. Let us hope, that the Cartwright lectures will take rank alongside the Gulstonian, Linnaean and other lecture courses, which have done so much for English medical science and literature. The committee were fortunate in their selection of a lecturer to inaugurate the course. Dr. Bartholow has given us a series of six lectures, in which he has collected the various contributions to our knowledge on this important topic of the antagonism of therapeutical agents, and has added not a little thereto as the result of his own experiments and study upon the subject. The book should be read by every scholarly physician.

